# Systematic review and meta-analysis of risk prediction models for anti-tuberculosis drug-induced liver injury in East Asian populations

**DOI:** 10.3389/fpubh.2026.1958154

**Published:** 2026-09-11

**Authors:** Hongjiao Li, Xin Feng, Xi Zheng, Xianmei Zhong

**Affiliations:** 1 Department of Pharmacy, Nanbu People's Hospital, Nanchong, China; 2 Department of Pulmonary and Critical Care Medicine, Nanbu People's Hospital, Nanchong, China; 3 Department of Pharmacy, Sichuan Academy of Medical Sciences & Sichuan Provincial People's Hospital, School of Medicine, University of Electronic Science and Technology of China, Chengdu, China

**Keywords:** anti-tuberculosis drug-induced liver injury, ATB-DILI, meta-analysis, prediction model, system evaluation

## Abstract

**Objective:**

This study systematically evaluates the construction methods, predictive factors, and performance of risk prediction models for anti-tuberculosis drug-induced liver injury (ATB-DILI). It aims to furnish evidence-based evidence for early detection of East Asian individuals at high risk of ATB-DILI and the development of personalized clinical treatment plans.

**Methods:**

A comprehensive literature search was conducted to identify studies related to risk prediction models for ATB-DILI. Key data, including study population, sample size, model construction algorithms, predictive factors, and model discrimination, were extracted. Qualitative synthesis and systematic analysis were then performed.

**Results:**

A total of 26 studies comprising 31 risk prediction models (cumulative sample size approximately 41,734 cases) were included. Twenty studies reported internal validation and six reported external validation. Model discriminative ability varied from 0.624 to 1.0, indicating at least modest-to-excellent discriminatory ability. Prediction model Risk Of Bias ASsessment Tool (PROBAST) assessment identified only one study at low risk of bias, with all remaining studies rated high risk of bias. Meta-analysis yielded a pooled AUC of 0.81 for ATB-DILI prediction models. History of liver disease, extrapulmonary tuberculosis, age ≥60 years, alcohol consumption, concomitant medication use, retreatment, elevated AST, diabetes, and smoking were associated with higher ATB-DILI risk (all *P* < 0.05), whereas uric acid showed an inverse association (*P* < 0.05). Importantly, the observed association for non-use of hepatoprotective agents cannot confirm a preventive effect; these factors should inform risk stratification and intensified monitoring rather than guide prophylactic hepatoprotective therapy.

**Conclusion:**

Published risk prediction models for ATB-DILI among East Asian populations were systematically appraised. These models yielded moderate-to-good discriminative performance yet showed substantial heterogeneity and high risk of bias, mainly due to defective missing-data processing, univariate-based predictor selection, insufficient outcome events, and absent external validation. Such methodological shortcomings may overestimate AUC values, especially for retrospective non-validated models. Existing ATB-DILI prediction models are thus of uncertain reliability and generalizability. Future large-sample multicenter prospective studies with standardized outcomes and external validation are required to develop robust clinically applicable models.

**Systematic review registration:**

https://www.crd.york.ac.uk/PROSPERO/view/CRD420261468400, identifier CRD420261468400.

## 1 Introduction

According to the latest World Health Organization (WHO) data, Tuberculosis (TB) was the world's leading cause of death from a single infectious agent in 2024, with an estimated 1.23 million deaths (1). The occurrence rate of TB in China remains persistently high, imposing a heavy burden on both the socioeconomic structure and the healthcare system (2). Although the efficacy of standard anti-TB treatment (e.g., the combination regimen of isoniazid, rifampicin, pyrazinamide, and ethambutol) is well established, the adverse drug reactions induced by this therapy should not be overlooked. Among these, anti-tuberculosis drug-induced liver injury (ATB-DILI) is the most common and most severe complication, with an incidence rate ranging from 9.5% to 14.1% in China (3). ATB-DILI can not only lead to treatment interruption or failure and the emergence of drug-resistant TB, but also, in severe cases, provoke acute liver failure and become life-threatening (4). Owing to the complex pathophysiology of ATB-DILI (involving direct hepatotoxicity of drugs and their metabolites, oxidative stress, idiosyncratic immune reactions, and host factors) and its nonspecific clinical manifestations, early diagnosis is extremely challenging (5–7). Therefore, the construction of a scientific and precise risk prediction model, enabling the identification of high-risk patients before or in the early phase of treatment, is of profound clinical significance for optimizing treatment plans and implementing preventive hepatoprotective measures.

A clinical prediction model is a statistical model that incorporates multiple patient characteristics (e.g., demographic features, medical history) to predict the occurrence of a certain outcome (8). Model development generally follows a fixed workflow, including pre-specified study design, variable screening, model fitting, internal validation, and external validation (8). This process can be implemented using traditional regression approaches, such as logistic regression, which assume a pre-defined functional form and offer high interpretability (9), or machine learning algorithms, such as random forest and extreme gradient boosting (XGBoost), which are more adept at capturing non-linearity, variable interactions, and high-dimensional features, but are less interpretable and more prone to overfitting (10). After model construction, performance is evaluated through discriminative ability (distinguishing patients with and without the event), calibration (agreement between predicted and actual risks), and external validation (ensuring validity in new settings) (8). This article aimed to systematically review the risk prediction models for ATB-DILI published in recent years, with a focus on model development strategies, risk of bias, and applicability analysis, to highlight the current methodological limitations and to define methodological requirements for future reliable models.

## 2 Data and methods

### 2.1 Inclusion and exclusion criteria

#### 2.1.1 Inclusion criteria

(1) The study included TB patients who were subjected to anti-TB treatment; (2) The study outcome was the occurrence of ATB-DILI; (3) A risk prediction model was developed.

#### 2.1.2 Exclusion criteria

(1) Animal- or cell-based experiments, case reports, reviews, and conference abstracts; (2) Key data required for model construction could not be obtained; (3) Only factors influencing the occurrence of ATB-DILI were analyzed, without involving or describing the construction of a prediction model.

### 2.2 Retrieval strategy

A computerized search of CNKI, Wanfang Data, VIP Database, Chinese Medical Journals network, OVID, PubMed, and EBSCO was conducted from database inception to August 15, 2026. A combination of subject headings and free-text words was used as the search strategy. The search terms included: “Anti tuberculosis drugs,” “liver injury,” “hepatitis,” “risk prediction,” “prediction model,” “nomogram,” and “machine learning.” The search strategy for PubMed is presented as an example (see Table 1).

**Table 1:** PubMed retrieval strategy.

Step	Search query
#1	(“Anti tuberculosis drugs”[MeSH Terms]) OR (Anti tuberculosis drugs [Title/Abstract])
#2	(“liver injury”[MeSH Terms]) OR (“hepatitis”[MeSH Terms]) OR (“liver injury”[Title/Abstract]) OR (hepatitis [Title/Abstract])
#3	(“prognosis”[MeSH Terms]) OR (“risk assessment”[MeSH Terms]) OR (“prediction model”[Title/Abstract]) OR (“risk score”[Title/Abstract]) OR (nomogram [Title/Abstract]) OR (“machine learning”[Title/Abstract])
#4	#1 AND #2 AND #3

### 2.3 Literature screening and data extraction

To ensure the objectivity of the study, literature screening and data extraction were independently performed by two researchers. Disagreements were resolved through discussion with a third reviewer. After the screening was completed, relevant data were extracted in accordance with the Checklist for Critical Appraisal and Data Extraction for Systematic Reviews of Prediction Modeling Studies (CHARMS). The extracted information included the first author, publication year, study design, sample size, characteristics of the study population, candidate predictors, modeling methods, model performance measures (discrimination), validation status, and model presentation format (11).

### 2.4 Model quality assessment

Two researchers independently evaluated the risk of bias and applicability of the included studies using the Prediction model Risk Of Bias ASsessment Tool (PROBAST) (12). Disagreements were resolved through consultation with a third reviewer. The PROBAST tool comprises four domains: participants, predictors, clinical outcomes, and analysis. Each domain is rated as “low risk,” “high risk,” or “unclear” based on signaling questions. The overall risk of bias is determined by the ratings of the individual domains; if any domain is rated as high risk, the overall risk of bias is considered high. The assessment of applicability focuses on three domains: participants, predictors, and clinical outcomes (13).

### 2.5 Statistical analysis

Meta-analysis was performed using Stata 18.0 software. The pooled AUC of the ATB-DILI prediction model was reported as the effect estimate with a 95% confidence interval (95% CI). Effect estimates for predictive factors were expressed as odds ratios (OR) with 95% CIs. Heterogeneity across studies was assessed using the I^2^ statistic. When I^2^ < 50% and *P* > 0.1, heterogeneity was considered low and a fixed-effects model was used. If I^2^ ≥ 50% or *P* ≤ 0.1, heterogeneity was considered substantial; sensitivity analysis was performed by sequentially omitting each study, and a random-effects model was employed if heterogeneity persisted. In addition, subgroup analyses were performed to identify potential sources of heterogeneity across AUC values, namely study model type, validation method, validation center size, events per variable (EPV), incidence of ATB-DILI, formal causality assessment for ATB-DILI definition and biochemical threshold for ATB-DILI definition.

## 3 Results

### 3.1 Literature screening process and results

**Figure 1:**
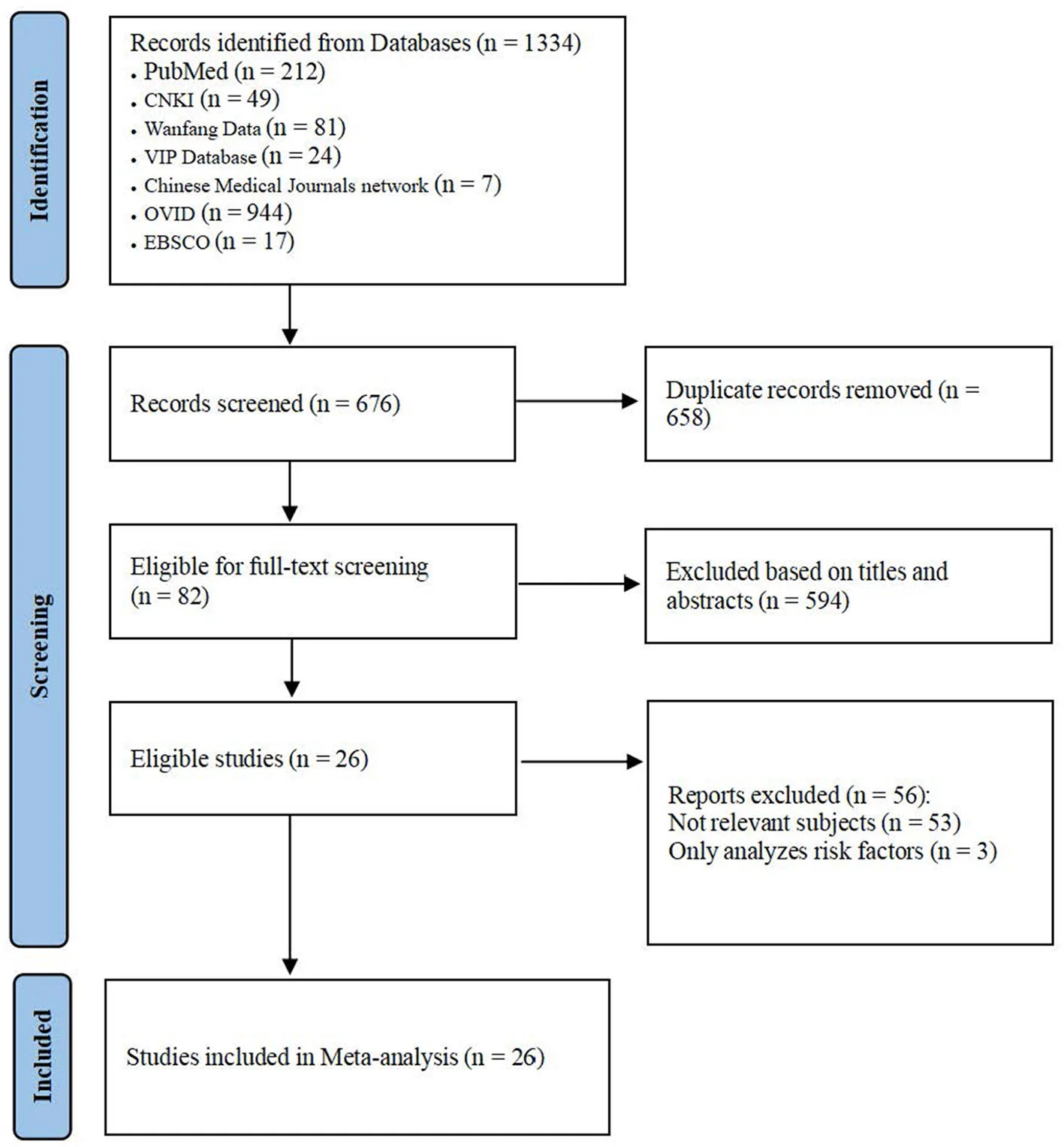
Flowchart of literature screening.

Total of 1,334 relevant records were retrieved in the initial search. After stepwise screening by reading titles and abstracts, removing duplicates, and reviewing full texts, 26 articles were ultimately included in the systematic review (14–39). The literature screening process is shown in Figure 1.

### 3.2 Basic characteristics of the included studies

This systematic review included 26 studies, of which 25 were retrospective studies conducted in China (14–33, 35–39) and one was a retrospective study conducted in Japan (34). The research population was all East Asians. The publication years ranged from 2016 to 2026. The total sample sizes ranged from 72 to 11,361 patients, and the number of incident ATB-DILI events varied from 19 to 3,787. The basic characteristics of the included studies are detailed in Supplementary Table 1.

### 3.3 Construction of prediction models

The 26 included studies reported a total of 31 prediction models. Regarding modeling methods, logistic regression (including multiple logistic regression) was used 18 times (14–18, 21–24, 27, 28, 30, 31, 34–38), Cox regression was used 4 times (19, 20, 29, 39), and neural networks (including convolutional neural networks, fully connected deep neural networks, etc.) were used 4 times (15, 25, 32, 39). In terms of ensemble learning, Random Forest and its variants (including distributed random forest and extremely randomized trees) were employed 6 times (26, 27, 32, 33, 36, 39). In addition, methods such as decision tree, support vector machine (SVM), k-nearest neighbors (KNN), naive Bayes, least absolute shrinkage and selection operator (LASSO), generalized linear model (GLM), and stacking ensemble were each applied one to two times. Variable selection approaches were diverse, primarily including univariate analysis, multivariable logistic regression, LASSO regression, and feature importance assessment based on machine learning. For missing data, multiple imputation, mean imputation, mode imputation, and median imputation were adopted in 6 studies (18, 26, 27, 36, 37, 39), whereas the remaining 20 studies either did not explicitly report the handling strategy or directly excluded cases with missing values. The number of predictors included in the final models ranged from 1 to 10. Details are presented in Supplementary Table 2.

### 3.4 Performance of the prediction models

For all models for which discrimination was reported, the AUC exceeded 0.6, a value indicative of at least modest discriminatory ability. Among these, three studies reported models with AUC values above 0.9(16, 21, 27). Calibration methods were reported in 14 studies, with results suggesting acceptable calibration (14, 16–18, 20–24, 28, 35, 37–39). Regarding validation, internal validation was performed in 21 studies (14–18, 20–28, 32, 33, 35–39), and external validation was conducted in 6 studies (18, 20, 22, 35, 36, 38). With respect to model presentation, nomograms were provided in 14 studies (14, 16–18, 20–22, 24, 26, 28, 31, 35, 37, 38); among them, the model presented by Gu et al. was a web-based nomogram (22), which offers greater convenience and speed. Regression model equations were presented in 5 studies (15, 20, 30, 34, 37), and the method of model presentation was not reported in 9 studies (19, 23, 25, 27, 29, 32, 33, 36, 39). Details are provided in Supplementary Table 3.

### 3.5 Risk of bias and applicability assessment

#### 3.5.1 Risk of bias assessment

**Figure 2:**
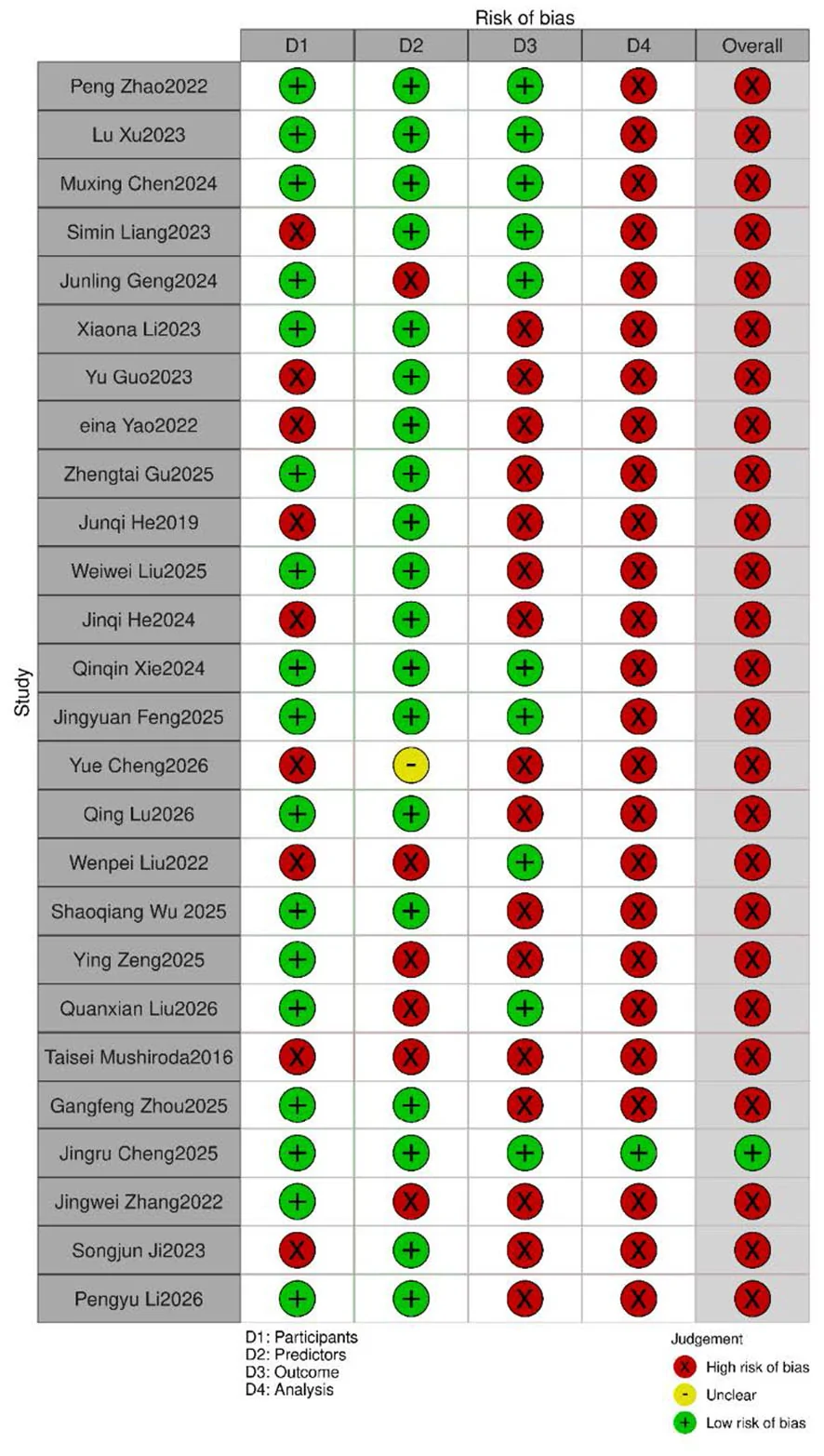
Risk of bias evaluation.

Among the 26 included studies, only one was judged to have an overall low risk of bias, while the remaining 25 were rated as having an overall high risk of bias (see Figure 2). In the domain of participants, 9 studies were at high risk of bias; in the domain of predictors, 6 studies were at high risk of bias; in the domain of clinical outcomes, 16 studies were at high risk of bias; and in the domain of analysis, 25 studies were at high risk of bias. Specifically: (1) Participants: 17 studies were at low risk of bias (14–16, 18, 19, 22, 24, 26, 27, 29, 31–33, 35–37, 39); 9 studies were at high risk of bias (17, 20, 21, 23, 25, 28, 30, 34, 38), among which 7 employed a non-nested case-control design (17, 21, 23, 25, 28, 30, 34), 3 had inappropriate inclusion and exclusion criteria (20, 23, 38), and 1 did not report the inclusion and exclusion criteria (34). (2) Predictors: 19 studies were at low risk of bias (14–17, 19–27, 29, 31, 35, 36, 38, 39); 6 studies were at high risk of bias, primarily because predictor information may not be obtainable when the model is intended for prediction (telomere length and single nucleotide polymorphisms in the study by Wenpei et al. (30) are not routinely available in clinical practice; rifampicin plasma concentration included in the study by Zeng et al. (32) is not accessible in some hospitals; metabolomics and microbiome data in the study by Liu et al. (33) are extremely difficult to obtain clinically; genotypes included in the studies by Junling et al. (18), Mushiroda et al. (34), and Zhang et al. (37) are not routinely available in clinical settings; 1 study was rated as unclear because predictor definitions were not reported (28). (3) Clinical outcomes: 16 studies were judged to be at high risk of bias (19–25, 28, 29, 31, 32, 34, 35, 37, 38), and 10 at low risk of bias (14–18, 26, 27, 30, 33, 36). The major contributing factors were the absence of formal causality assessment in 14 studies (19, 20, 22–25, 28, 31, 32, 34, 35, 37–39) and lack of reference guidelines in 4 studies (20, 21, 23, 38) (see Supplementary Table 4 for details). (4) Analysis: Only 1 study was at low risk of bias (36), and the remaining 25 were at high risk of bias. The main reasons were: 12 studies had an EPV < 10 or the number of outcome events in the validation was < 100, and 8 studies had an unclear EPV or unclear number of outcome events in the validation; 21 studies did not describe the handling of missing data; only 6 studies conducted external validation (18, 20, 22, 35, 36, 38), 21 performed internal validation (14–18, 20–28, 32, 33, 35–39), and 5 lacked either internal or external validation (19, 29–31, 34). In 20 studies, predictors were screened by univariate analysis before being entered into the multivariable model (14–25, 28–32, 35, 38, 39).

#### 3.5.2 Applicability assessment

**Figure 3:**
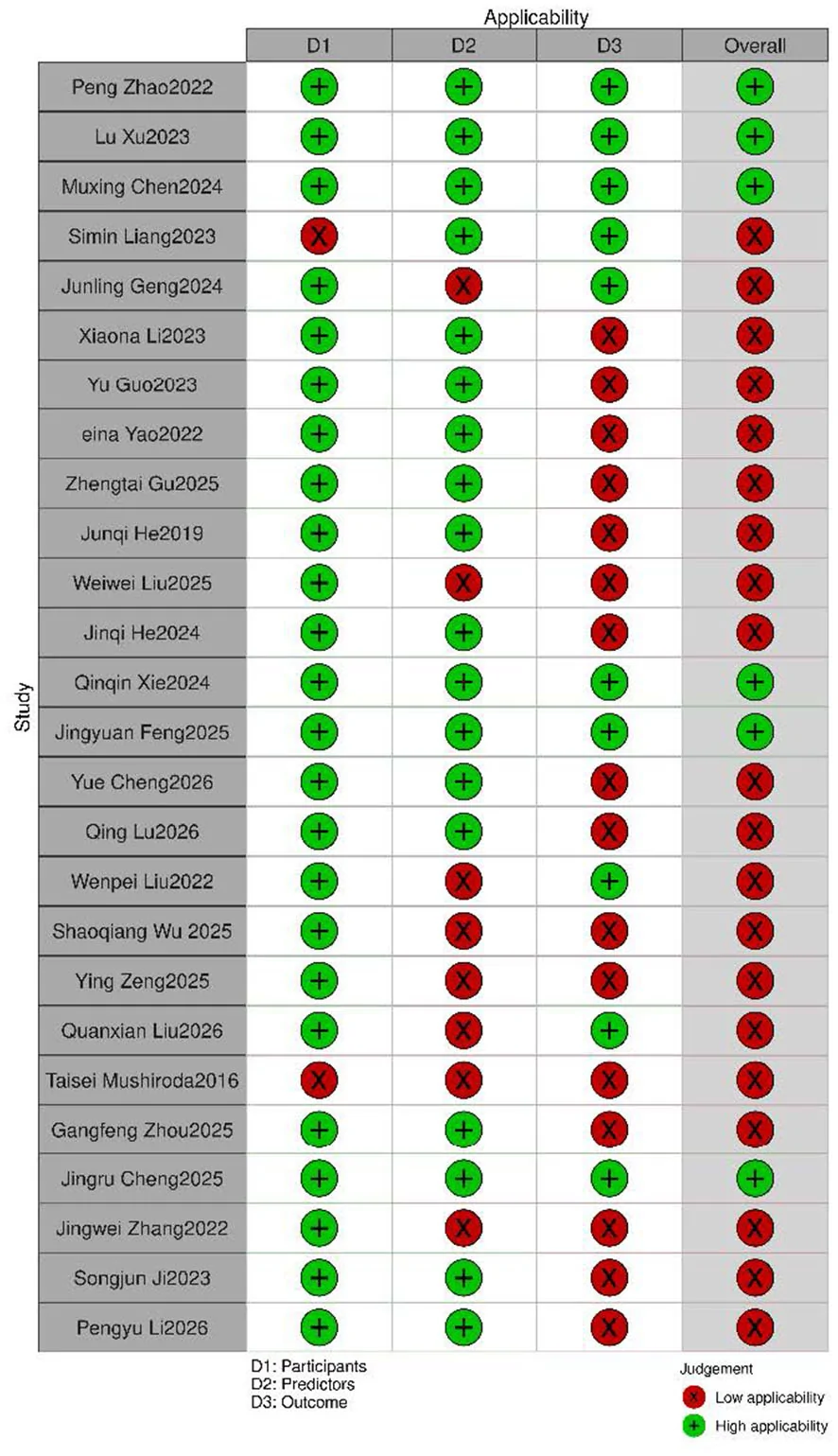
Applicability evaluation.

For applicability, 6 studies (14–16, 26, 27, 36) were judged to have high overall applicability and 20 studies (17–25, 28–35, 37–39) low overall applicability (Figure 3). Specifically: (1) Study population: low applicability was identified in 2 studies; one included only Uyghur individuals from southern Xinjiang (17), and the other was conducted in a Japanese population (34). (2) Predictors: low applicability was observed in 8 studies; among these, one required identification of Traditional Chinese Medicine body constitution (24), one required telomere/single nucleotide polymorphism assessment (30), one required FibroScan (31), one required multi-omics analysis (33), one required therapeutic drug monitoring of blood concentrations (32), and three required genetic testing (18, 34, 37). (3) Clinical outcomes: Low applicability in 16 studies was mainly attributable to the lack of formal causal effect assessment (14 studies) (19, 20, 22–25, 28, 31, 32, 34, 35, 37–39) and the absence of reference-based clinical outcome guidelines (4 studies) (20, 21, 23, 38).

### 3.6 Meta-analysis results

#### 3.6.1 AUC analysis

A total of 26 studies on prediction models for ATB-DILI were included. Among them, 13 studies reported the AUC and its 95% confidence interval (CI) (14, 16–19, 21, 22, 24, 28, 30, 33, 35, 39), while the remaining 13 studies did not report 95% CI values (15, 20, 23, 25–27, 29, 31, 32, 34, 36–38) and were therefore excluded from the meta-analysis of AUC synthesis.

**Figure 4:**
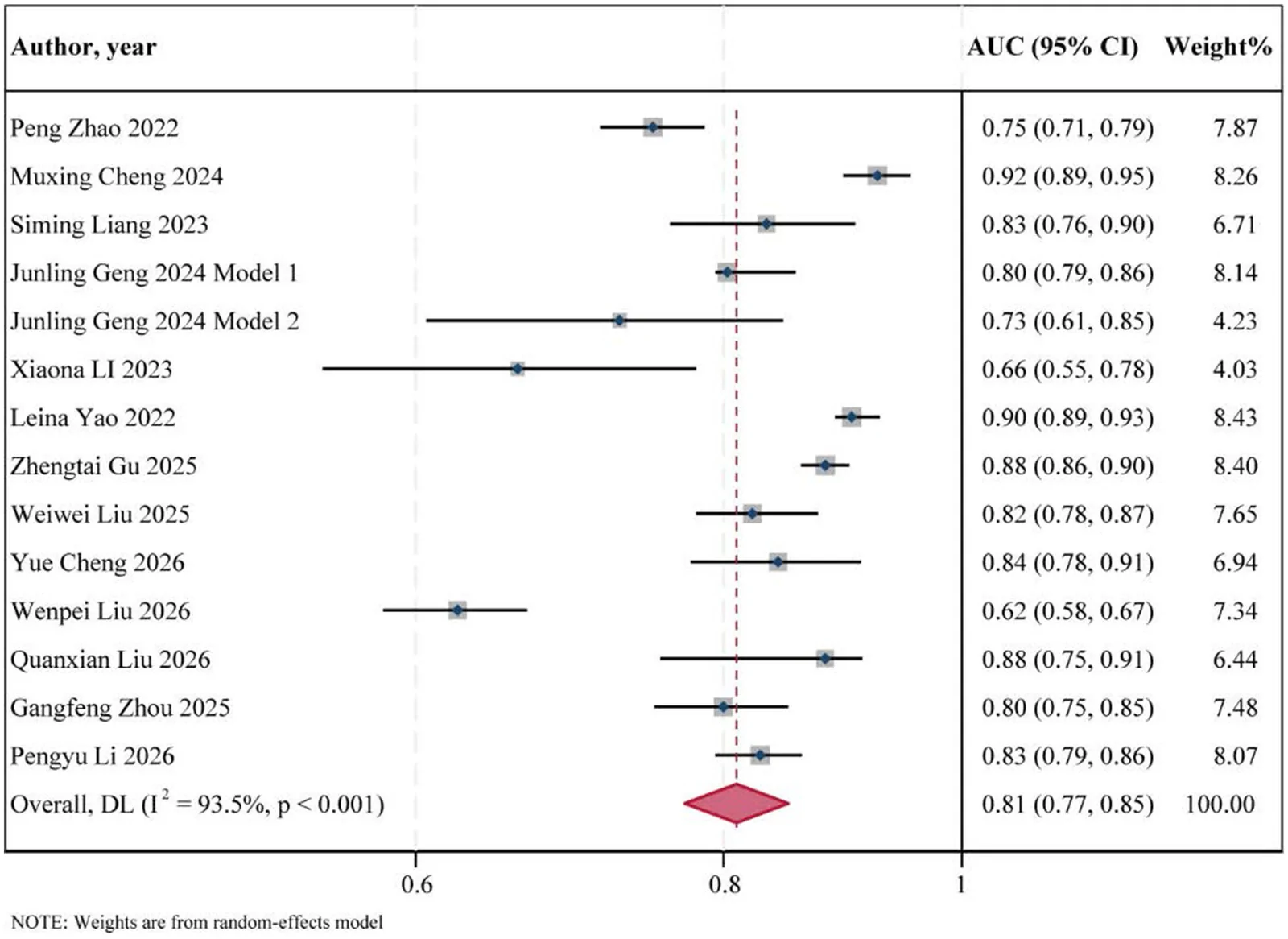
Forest plot of the AUC value for the prediction model of drug-induced liver injury from anti-tuberculosis drugs.

The heterogeneity test for the meta-analysis of pooled AUCs demonstrated substantial between-study heterogeneity (I^2^ = 93.5%, *P* < 0.001). Sensitivity analysis of AUC estimates showed that pooled results remained stable following sequential exclusion of individual studies. Given the high heterogeneity, a random-effects model was applied for quantitative pooling. The pooled AUC under the random-effects model was 0.81 (95% CI: 0.77–0.85), yet high heterogeneity persisted (Figure 4); accordingly, we conducted subgroup analyses to identify potential sources of heterogeneity across included studies. Subgroup analyses (Table 2) indicated that machine-learning models yielded numerically higher pooled AUC values compared with traditional regression models: *n* = 2, pooled AUC = 0.84 (95% CI: 0.80–0.88) vs. *n* = 12, pooled AUC = 0.80 (95% CI: 0.76–0.85). Models lacking external validation had a lower pooled AUC of 0.65 (95% CI: 0.60–0.71). Single-center models produced a pooled AUC of 0.84 (95% CI: 0.80–0.88), which exceeded that of multi-center models (0.76, 95% CI: 0.68–0.85). The pooled AUC for studies adopting consistent biochemical thresholds to define ATB-DILI was 0.77, lower than the pooled AUC of 0.88 among studies using other biochemical thresholds. Substantial residual heterogeneity was observed within most subgroups. Several subgroups contained merely two to three studies; thus, these subgroup comparisons were exploratory and did not provide conclusive findings.

**Table 2:** Results of subgroup analyses for pooled AUC.

Included studies	Number of prediction models	AUC (95%CI)	I^2^ (%)	*P* value
All studies	14	0.81 (0.77, 0.85)	93.5	< 0.001
**Model type**				
Traditional-regression algorithms	12	0.80 (0.76, 0.85)	94.4	< 0.001
Machine-learning algorithms	2	0.84 (0.8, 0.88)	25.75	0.246
**Validation method**				
No validation	3	0.65 (0.6, 0.71)	30	0.24
Internal validation	8	0.85 (0.8, 0.89)	90.4	< 0.001
Internal and external validation	3	0.83 (0.77, 0.89)	90.6	< 0.001
**Study-center size**				
Single-center	9	0.84 (0.8, 0.88)	90.3	< 0.001
Multi-center	5	0.76 (0.68, 0.85)	92.3	< 0.001
**Events per variable (EPV)**				
≥10	9	0.78 (0.73, 0.84)	95.0	< 0.001
< 10	2	0.88 (0.83, 0.94)	62.7	0.101
Not specified	3	0.82 (0.79, 0.86)	37.7	0.201
**Incidence of ATB-DILI**				
≥20%	5	0.78 (0.68, 0.90)	96.6	< 0.001
< 20%	6	0.84 (0.79, 0.88)	91.2	< 0.001
Not specified	3	0.81 (0.78, 0.83)	0.2	0.367
**Formal causality assessment for ATB-DILI definition**				
RUCAM score ≥ 3	8	0.81 (0.74, 0.87)	96.0	< 0.001
Not specified	6	0.83 (0.79, 0.87)	78.6	< 0.001
**Biochemical threshold for ATB-DILI definition**				
Consistent	9	0.77 (0.72, 0.83)	93.5	< 0.001
Other	5	0.88 (0.84, 0.92)	79.1	< 0.001

RUCAM, the Roussel Uclaf Causality Assessment Method.

#### 3.6.2 Predictor analysis

A total of 31 prediction models for ATB-DILI were included in this study. The number of initial candidate predictors ranged from 6 to 53, and the final models contained between 1 and 10 predictors. Of the 22 candidate predictors reported in ≥2 studies, 6 were excluded from the meta-analysis because of incomplete data and 4 owing to substantial discrepancies in predictor definitions. Pooled effect-size analyses were therefore conducted for 11 predictors: history of liver disease, extrapulmonary TB, age ≥60 years, alcohol consumption, non-use of hepatoprotective drugs, concomitant medication use, retreatment, uric acid, aspartate aminotransferase (AST), diabetes, and smoking (See Supplementary Table 5 for predictor definitions). Heterogeneity testing demonstrated substantial heterogeneity for history of liver disease, age ≥60 years, non-use of hepatoprotective drugs, retreatment, and smoking, whereas low heterogeneity was seen for extrapulmonary TB, alcohol consumption, concomitant medication use, uric acid, AST, and diabetes.

Pooled-effect analysis showed that a history of liver disease, extrapulmonary TB, age ≥60 years, alcohol consumption, concomitant medication use, retreatment, elevated AST, diabetes, and smoking were associated with increased ATB-DILI risk (all *P* < 0.05), whereas uric acid was inversely associated with ATB-DILI (*P* < 0.05). An association was also observed for non-use of hepatoprotective drugs, though this finding does not imply a causal preventive effect of hepatoprotective agents. Detailed results are presented in Table 3.

**Table 3:** Meta-analysis results on influencing factors of drug-induced liver injury from anti-tuberculosis drugs.

Predictors	Number of studies included	Merge effect value		Heterogeneity test	
		Random-effects model	Fixed-effects model	I^2^ (%)	*P* value
History of liver disease (categorical variables)	9	OR: 3.232 95% CI: 2.098–4.979 Z: 5.319 *P*: 0.000	——	81.6	0.929
Extrapulmonary tuberculosis (categorical variables)	5	——	OR: 2.236 95% CI: 1.823–2.741 Z: 7.738 *P*: 0.000	11.4	0.693
Age ≥ 60 years (categorical variables)	4	OR: 2.443 95% CI: 1.070–5.579 Z: 2.1220 *P*: 0.034	——	97.2.2	0.993
Alcohol consumption (categorical variables)	5	——	OR: 2.857 95% CI: 2.064–3.955 Z: 6.331 *P*: 0.000	27.4	0.741
No use of hepatoprotective agents (categorical variables)	2	OR: 1.611 95% CI: 1.219–2.130 Z: 3.351 *P*: 0.001	——	0.0	0.0
Concomitant medication use (categorical variables)	2	——	OR: 4.544 95% CI: 2.574–8.023 Z: 5.219 *P*: 0.000	35.4	0.871
Retreatment (categorical variables)	2	OR: 2.946 95% CI: 1.596–5.439 Z: 3.455 *P*: 0.001	——	0.0	0.0
Uric acid (continuous variables)	3	——	OR: 0.998 95% CI: 0.996–0.999 Z: −3.813 *P*: 0.000	0.0	0.331
AST (continuous variables)	2	——	OR: 1.032 95% CI: 1.018–1.046 Z: 4.447 *P*: 0.000	0.0	0.703
Diabetes mellitus (categorical variables)	2	——	OR: 2.096 95% CI: 1.516–2.898 Z: 4.476 *P*: 0.000	0.0	0.718
Smoking (categorical variables)	2	OR: 1.880 95% CI: 1.237–2.859 Z: 2.954 *P*: 0.003	——	0.0	0.0

——indicates that no relevant analysis has been conducted.

## 4 Discussion

This systematic review included 26 studies involving 31 risk-prediction models for ATB-DILI. In the meta-analysis of pooled AUC values, the summary AUC was 0.81, with substantial between-study heterogeneity (I^2^ = 93.5%, *P* < 0.001), indicating considerable discrepancies across included studies in model development, validation design, and clinical contexts. Notably, this pooled estimate was derived from only 14 prediction models that reported 95% confidence intervals and thus cannot represent the overall discriminative performance of all eligible models. To explore potential sources of heterogeneity, we conducted subgroup analyses based on pre-specified covariates. However, substantial residual heterogeneity remained evident in most subgroups. Additionally, several subgroups contained only 2–3 studies. Collectively, these findings render subgroup comparisons purely exploratory rather than conclusive. Crucially, included studies exhibited marked heterogeneity in ATB-DILI outcome definitions, encompassing inconsistent biochemical cut-offs, variable adherence to diagnostic guidelines, optional implementation of formal causality assessment tools (e.g., RUCAM), non-uniform exclusion of alternative hepatic injury etiologies, and diverse definitions of liver injury observation time windows. Such outcome definition heterogeneity, combined with the lack of external validation in most models and the high risk of bias assigned to 25 of 26 included studies, likely constitutes the primary underlying driver of the elevated I^2^ value.

Methodological quality is a critical factor influencing the reliability of prediction models. The present review found that the included studies were commonly at high risk of bias, primarily due to inappropriate handling of missing data (20/26), predictor selection based on univariate analysis (20/26), lack of external validation (20/26), non-standardized outcome definition or ascertainment methods (16/26 studies), insufficient sample size (12/26), and a non-nested case-control design (7/26). These findings suggest that the reported model performance metrics (e.g., high AUC values) may be largely optimistic, and that the discrimination and calibration of these models may decline substantially when applied to new, heterogeneous patient populations. Clinicians should therefore exercise caution when referring to these models.

Accumulating evidence demonstrates that factors associated with ATB-DILI risk have extended beyond conventional liver biochemical markers, age and nutritional status to novel domains such as genetic polymorphisms, multi-omics biomarkers, and traditional Chinese medicine constitutions. However, genetic polymorphisms and multi-omics testing incur high costs and lack sufficient standardization, whereas traditional Chinese medicine constitution assessment is highly subjective, which may limit their widespread implementation in clinical practice. In the pediatric prediction models from the present systematic review, drug-exposure metrics including peak rifampicin concentration and cumulative isoniazid dose emerged as predictors alongside routine liver-function parameters. This finding implies that therapeutic drug monitoring (TDM) may be implemented in children receiving anti-TB therapy.

Pooled effect size analysis of the 11 meta-analyzed predictors revealed significant associations. Two predictors—a history of liver disease and age ≥60 years—fall within the mechanistic framework of “baseline hepatic function and drug metabolism and clearance.” This observation indicates that impaired hepatic reserve and drug-clearance capacity constitute key biological pathways driving ATB-DILI. Patients with a history of liver disease, particularly chronic hepatitis B or C, commonly show decreased expression and activity of most CYP metabolic enzymes, which further decline with worsening hepatic fibrosis (40). This leads to reduced clearance of anti-TB drugs and their toxic metabolites, as well as increased systemic accumulation. The finding suggests that in regions with high endemicity of hepatitis B/C, history of liver disease should be used as the primary screening indicator for ATB-DILI risk stratification. Increasing age is accompanied by declines in hepatic and renal function, reductions in hepatic blood flow and liver volume, and deterioration of CYP metabolic enzyme capacity (41, 42), resulting in decreased drug clearance and increased exposure. Age is an independent predictor in this meta-analysis, consistent with age-related physiological deterioration. Several studies show that ATB-DILI risk rises substantially beyond 60 years of age (15, 28, 29).

The four factors—alcohol consumption, diabetes, smoking, and uric acid—can be categorized under the dimension of “exacerbated liver vulnerability and oxidative stress.” Long-term alcohol consumption markedly alters the expression and activity of multiple CYP450 enzymes, accompanied by fat accumulation and aggravated oxidative stress (43, 44). Diabetes can induce hepatic metabolic imbalance, thereby impairing the liver's drug clearance capacity and further potentiating drug-induced liver injury (45). Smoking exacerbates the hepatic burden through CYP1A2 induction and oxidative stress (46, 47). Uric acid is a profound endogenous antioxidant in the human body; the observed negative association between uric acid and ATB-DILI may be partially in response to lower endogenous antioxidant buffering capacity, as ATB-DILI itself is intimately linked to increased reactive oxygen species (ROS), lipid peroxidation, and depletion of antioxidant defense systems (5, 48).

The three factors—concomitant medication, retreatment, and non-use of hepatoprotective agents—were categorized into the dimension of “treatment-related exposure and drug interactions.” The incidence and severity of ATB-DILI are significantly elevated by the co-administration of other potentially hepatotoxic drugs with anti-TB therapy, through multiple mechanisms including induction and competition of hepatic drug-metabolizing enzymes, superimposition of toxicity pathways, and depletion of antioxidant defenses (49, 50). In retreatment patients, pre-existing impairment of liver function may have resulted from prior therapy, or drug-resistant strains may necessitate more complex treatment plans containing second-line drugs, thereby heightening the risk of hepatotoxicity (51, 52). Non-use of hepatoprotective drugs was significantly associated with elevated ATB-DILI risk. Nevertheless, this association does not infer a causal preventive effect of hepatoprotective drugs against ATB-DILI. Since this meta-analysis pools observational study data, the prescription of agents may be shaped by confounders such as baseline liver function, comorbidities, and clinical management decisions. Consequently, confounding by indication and residual confounding cannot be excluded.

Extrapulmonary TB emerged as an independent predictor in this meta-analysis. Its link with ATB-DILI should be interpreted in terms of clinical drug exposure and host pathological status. Extrapulmonary TB (including central nervous-system, osteoarticular and disseminated TB) generally requires prolonged anti-TB therapy. Disease complexity, drug-resistance risk and treatment interruptions increase cumulative hepatotoxic-drug exposure and regimen modifications, which elevates hepatic dysfunction risk (53–55). At the host pathophysiological level, extrapulmonary TB is associated with prominent systemic inflammation and metabolic consumption, along with longer hospitalization, substantial weight loss and increased risk of persistent malnutrition. Hypoalbuminemia (< 35 g/L), which indicates impaired hepatic reserve and high inflammatory burden, has been validated as a correlate of drug-induced liver injury in meta-analyses and prospective studies (56, 57). Mechanistically, oxidative stress from anti-TB drugs amplifies hepatocellular injury via NF-κB/NLRP3 signaling. Systemic inflammation in extrapulmonary TB may synergise with this pathway to heighten hepatic vulnerability to drug toxicity (58, 59). Extrapulmonary TB probably acts as a composite surrogate (severity, complexity, inflammation/nutrition) rather than a direct biological predictor. Future models should refine it into quantifiable mediators, and clinically, these patients need intensified liver monitoring beyond routine assessment.

This meta-analysis revealed that AST was associated with ATB-DILI risk among included prediction models. ALT could not be pooled owing to inconsistent definitions across studies, while the AST meta-analysis included merely two studies. Accordingly, this finding warrants cautious interpretation and does not constitute definitive evidence that ALT lacks predictive value for ATB-DILI. High-quality prospective studies are needed in future to validate the predictive performance of AST and ALT for ATB-DILI in diverse populations. Clinically, ALT and AST are used jointly as monitoring markers to define liver injury. Given that our analysis represents only a preliminary and limited exploration, its findings should not drive changes to existing clinical management strategies.

In terms of modeling, included studies adopted conventional logistic and Cox regression, as well as a broad set of machine-learning algorithms including neural networks, tree-based models, vector machines and stacking ensembles (15, 25–27, 32, 33, 36). Overall, machine-learning models yielded slightly higher discrimination of 0.84 (0.80-0.88) compared with 0.80 (0.76-0.85) for traditional regression models. Conventional regression relies on linear and additive assumptions, which may oversimplify real-world non-linear patterns, threshold-dependent exposure-response relationships, and complex interactions, limiting discriminative performance and introducing biased inferences (60). Machine-learning can automatically capture these nonlinearities to boost predictive capacity (61). Discrimination near 0.8 warrants caution; perfect performance often signals overfitting or data leakage, yielding inflated and non-reproducible results (62). Discrimination near 0.8 warrants caution; perfect performance often signals overfitting or data leakage, yielding inflated and non-reproducible results.

Several limitations of the present study should be acknowledged. First, our search strategy may not be sufficiently sensitive to capture studies indexed in non-East-Asian databases, leading to a study sample restricted exclusively to East-Asian populations. Consequently, our findings are not generalizable to other racial and ethnic groups. Second, more than 40% of studies were excluded from the pooled AUC meta-analysis; accordingly, the pooled AUC estimate may be subject to selection bias and might not reflect the overall performance across all ATB-DILI prediction models. Third, while the AUC quantifies discriminative ability, only a small number of studies performed external validation. Accordingly, these summary estimates should not be interpreted as the performance achievable in real-world clinical settings.

## 5 Conclusion

This study systematically appraised published risk prediction models for ATB-DILI among East Asian populations. While the included models achieved moderate-to-good discriminative performance, substantial heterogeneity across studies was observed. Collectively, these models were associated with high overall risk of bias, predominantly stemming from analytical shortcomings. Major methodological concerns encompassed inappropriate or incompletely reported missing-data management, predictor selection based on univariate analyses, inadequate numbers of outcome events (violations of the events-per-variable criterion), and the absence of external validation. These methodological flaws are likely to inflate reported AUC values. Retrospectively derived models without sufficient validation are particularly prone to such optimistic performance bias. Accordingly, the reliability and external generalizability of existing models remain unclear. Future large-sample, multicenter prospective cohort studies are urgently warranted: these should implement standardized outcome definitions, apply appropriate missing-data approaches, eschew predictor screening exclusively by univariate analysis, conduct rigorous assessment of model performance metrics, and incorporate external validation. These methodological improvements are indispensable for developing robust, transportable risk prediction models and enabling their eventual implementation in real-world clinical settings.

## Data Availability

The original contributions presented in the study are included in the article/Supplementary material, further inquiries can be directed to the corresponding author.
