# Supplementary material for: Systematic review and meta-analysis of risk prediction models for anti-tuberculosis drug-induced liver injury in East Asian populations
Source: Front Public Health. 2026 Sep 11;14:1958154. doi: 10.3389/fpubh.2026.1958154 (PMC13612468; doi:10.3389/fpubh.2026.1958154)
Supplement: Supplementary file 1 [file Table_1.docx]

**Supplementary Materials**

**Table 1 Basic characteristics of included literature**

| **Study** | **Publication Date** | **Case collection time** | **Research location** | **Model Type** | **Research design** | **Research subject** | **Data source** | **Total sample size** | **Number of outcome events** | **Incidence** | **Outcome** |
| --- | --- | --- | --- | --- | --- | --- | --- | --- | --- | --- | --- |
| Peng Zhao | 2022 | 2017-2021 | 1 hospital in Guiyang | E+V1 | Cohort study | Patients on anti-TB treatment | EHR | 5681 | 214 | 3.8% | ATB-DILI |
| Lu Xu | 2023 | 2019-2022 | 3 hospitals in Jiangsu | E+V1 | Cohort study | Patients on anti-TB treatment | EHR | 3012 | 294 | 9.76% | ATB-DILI |
| Muxing Chen | 2024 | 2019-2023 | 1 hospital in Fuzhou | E+V1 | Cohort study | Patients on anti-TB treatment | EHR | 464 | 232 | 50% | ATB-DILI |
| Simin Liang | 2023 | 2020-2022 | 1 hospital in Xinjiang | E+V1 | Case-controlstudy | Patients on anti-TB treatment | EHR | 300 | 41 | 13.67% | ATB-DILI |
| Junling Geng | 2024 | 2017-2023 | 2 hospitals in Jiangsu | E+V1,2; E | Cohort study | Patients on anti-TB treatment | EHR | 498, 330 | — | — | ATB-DILI |
| Xiaona Li | 2023 | 2011-2021 | 1 hospital in Qingdao | E | Cohort study | Patients on anti-TB treatment | Questionnaire, EHR | 90 | 30 | 33.33% | ATB-DILI |
| Yu Guo | 2023 | 2021-2021 | 1 hospital in Shijiazhuang | E+V1,2 | Cohort study | Patients on anti-TB treatment | HER | 369 | 74 | 20.05% | ATB-DILI |
| Leina Yao | 2022 | 2018-2019 | 1 hospital in Fuyang | E+V1 | Case-controlstudy | Patients on anti-TB treatment | HER | 1160 | 60 | 5.17% | ATB-DILI |
| Zhengtai Gu | 2025 | 2019-2022 | 1 hospital in Fuyang | E+V1,2 | Cohort study | Patients on anti-TB treatment | EHR | 1396 | 140 | 10.03% | ATB-DILI |
| Junqi He | 2019 | 2015-2016 | 2 hospitals in Shenzhen | E+V1 | Case-controlstudy | Patients on anti-TB treatment | EHR | 208 | 34 | 16.35% | ATB-DILI |
| Weiwei Liu | 2025 | 2021-2025 | 1 hospital in Rufu | E+V1 | Cohort study | Patients on anti-TB treatment | EHR | 1393 | — | — | ATB-DILI |
| Jinqi He | 2024 | 2019-2019 | 1 hospital in Hulunbuir | E+V1 | Case-controlstudy | Patients on anti-TB treatment | EHR | 158 | 71 | 44.94% | ATB-DILI |
| Qinqin Xie | 2024 | 2013-2022 | 1 hospital in Chongqing | E+V1 | Cohort study | Patients on anti-TB treatment | EHR | 2796 | 153 | 5.47% | ATB-DILI |
| Jingyuan Feng | 2025 | 2018-2022 | 1 hospital in Guangzhou | E+V1 | Cohort study | Patients on anti-TB treatment | EHR | 11361, 1017 | 3787, 339 | 33.33% | ATB-DILI |
| Yue Cheng | 2026 | 2021-2023 | 1 hospital in Anhui | E+V1 | Case-controlstudy | Patients on anti-TB treatment | EHR | 233 | 35 | 15.02% | ATB-DILI |
| Qing Lu | 2026 | 2015-2024 | 1 hospital in Taicang | E | Cohort study | Patients on anti-TB treatment | EHR | 522 | 75 | 14.23% | ATB-DILI |
| Wenpei Liu | 2022 | 2016-2019 | 4 hospitals in Jiangsu | E | Case-controlstudy | Patients on anti-TB treatment | EHR | 1010 | 202 | 20% | ATB-DILI |
| Shaoqiang Wu | 2025 | 2022-2023 | 1 hospital in Hunan | E | Cohort study | Patients on anti-TB treatment | EHR | 133 | 65 | 48.87% | ATB-DILI |
| Ying Zeng | 2025 | 2020-2024 | 1 hospital in Changsha | E+V1 | Cohort study | Patients on anti-TB treatment | EHR | 184 | 19 | 10.33% | ATB-DILI |
| Quanxian Liu | 2026 | 2022-2023 | 2 hospitals in Sichuan and 1 hospital in Zunyi | E+V1 | Cohort study | Patients on anti-TB treatment | EHR | 72 | 35 | 48.61% | ATB-DILI |
| Taisei Mushiroda | 2016 | — | 1 hospital in Japan | E | Case-controlstudy | Patients on anti-TB treatment | EHR | 366 | 73 | 19.95% | ATB-DILI |
| Gangfeng Zhou | 2025 | 2022-2024 | 1 hospital in Ganzhou and 1 hospital in Fuzhou | E+V1,2 | Cohort study | Patients on anti-TB treatment | EHR | 2724 | 137 | 5.03% | ATB-DILI |
| Jingru Cheng | 2025 | 2017-2024 | 4 hospitals in China | E+V1,2 | Cohort study | Patients on anti-TB treatment | EHR | 3279 | 546 | 16.65% | ATB-DILI |
| Jingwei Zhang | 2022 | 2016-2018 | 1 hospital in Sichuan | E+V1 | Cohort study | Patients on anti-TB treatment | EHR | 746 | 118 | 15.82% | ATB-DILI |
| Songjun Ji | 2023 | 2020-2022 | 1 hospital in Wenzhou | E+V1,2 | Cohort study | Patients on anti-TB treatment | EHR | 1979 | — | — | ATB-DILI |
| Pengyu Li | 2026 | 2019-2026 | 1 hospital in Changsha | E+V1 | Cohort study | Patients on anti-TB treatment | EHR | 253 | 54 | 21.34% | ATB-DILI |

**Note:** HER = Electronic Health Record; ATB‑DILI = anti‑tuberculosis drug‑induced liver injury. E = denotes model development; E+V1 = model development with internal validation; E+V1,2 = model development with internal and external validation. — = indicates that the data were not reported.

**Table 2 Construction of a risk prediction model for drug-induced liver injury in tuberculosis patients**

| **Study** | **Modeling method** | **Variable selection** | **Number of candidate variables** | **Sample size (D/I/E)** | **Processing of missing data** | **Final predictive factor** | **Number of predictive factors** | **Events per variable** |
| --- | --- | --- | --- | --- | --- | --- | --- | --- |
| Peng Zhao | LR | Univariate + multivariate LR | 17 | — | — | Tuberculosis type, malnutrition, concomitant underlying liver disease, intermittent use of hepatoprotective agents, full‑course use of hepatoprotective agents. | 5 | 214/5=42.8 |
| Lu Xu | Convolutional neural network and multiple logistic regression | Convolutional neural network, univariate + multivariate LR | 53 | 2108/904/— | — | Model 1: age, history of liver disease, hypoalbuminemia, no prophylactic use of hepatoprotective agents; Model 2: Model 1 plus alcohol consumption. | 5，4 | 294/5=58.8 |
| Muxing Chen | LR | Univariate + multivariate LR | 14 | 324/140/— | — | Extrapulmonary tuberculosis, concomitant medications, alcohol abuse, HBsAg positivity, underlying liver disease history, age, serum albumin, hemoglobin, BMI. | 9 | 232/9≈25.78 |
| Simin Liang | LR | Univariate + multivariate LR | 11 | — | — | Concomitant extrapulmonary tuberculosis, retreatment, hypoproteinemia, underlying liver disease history. | 4 | 41/4=10.25 |
| Junling Geng | LR | Univariate + multivariate LR | Model 1: 19;  Model 2: 6 | Model 1: 348/150/—;  Model 2: 330/—/— | Model 1: Multiple imputation; Model 2: — | Model 1: extrapulmonary tuberculosis, INR, UA, HGB, PLT, blood glucose; Model 2: ABCB6 gene rs1109866, rs3731885; ABCG2 gene rs2231142, rs3114018, rs2622605. | Model 1: 6; Model 2: 5 | Model 1: —;  Model 2: 10/5=22 |
| Xiaona Li | Cox regression | LR | 10 | 90/—/— | — | Succinic acid (succinate). | 1 | 30/1=30 |
| Yu Guo | Cox regression | Random Forest importance scores with LASSO regression | 23 | 297/72/— | — | AST, DBIL, CYP2E1, NAT2, MDH. | 5 | 74/5=14.8 |
| Leina Yao | LR | Bivariate + multivariate LR | 27 | 1160/—/— | — | Age, underlying liver disease, diabetes mellitus, hepatoprotective agents, albumin, TBIL, pyrazinamide‑containing regimen, concomitant use of other hepatotoxic drugs. | 8 | 60/8=7.5 |
| Zhengtai Gu | LR | Univariate + multivariate LR | 24 | 872/524/— | — | Age, diabetes mellitus, smoking, liver‑related diseases, ALB, AST, GGT, TBil. | 8 | 140/8=17.5 |
| Junqi He | LR | Univariate + multivariate LR | 10 | 156/52/— | — | Model 1 (living environment + working environment), Model 2 (Model 1 + diagnosis), Model 3 (Model 2 + age). | 2，3，4 | 52/4=13 |
| Weiwei Liu | LR | Univariate + multivariate LR | 25 | 978/415/— | — | Electrolyte disturbances, underlying chronic liver disease, history of alcohol consumption, Qi‑deficiency constitution, Damp‑Heat constitution, Phlegm‑Dampness constitution. | 6 | — |
| Jinqi He | Neural network model | Univariate analysis + neural network model | 11 | 111/47/— | — | Age, average sleep duration. | 2 | 71/2=35.5 |
| Qinqin Xie | Extreme Gradient Boosting, Adaptive Boosting, Light Gradient Boosting, Random Forest | Univariate analysis and LASSO regression | 28 | 1957/839/— | Multiple imputation | Treatment history (retreatment), baseline ALT, baseline GGT, length of hospital stay, days of isoniazid treatment, cumulative total dose of isoniazid, baseline DBIL, days of ethambutol treatment. | 8 | 153/8=19.125 |
| Jingyuan Feng | Adaptive Boosting, Decision Tree, Gradient Boosting Machine, K‑Nearest Neighbors, Least Absolute Shrinkage and Selection Operator, Logistic Regression, Naive Bayes, Random Forest, Support Vector Machine, Extreme Gradient Boosting | Machine learning feature importance analysis | 34 | Model 1: 5680/5680/—; Model 1: 508/508/— | Mean | Model 1: ALT, EOS, AST, PCT, PLT, MLR, FIB, DBIL, HGB, BUN; Model 2: TBA, PCT, ALP, ChE, PA, CRP, ALT, AST, GGT, CD4/CD8. | 10 | Model 1: 3787/10=378.7，Model 2: 339/10=33.9 |
| Yue Cheng | LR | Importance analysis | 17 | 233/—/— | — | Age, BMI, nutritional status, alcohol consumption, whether a new case (initial patient), underlying liver disease. | 6 | 35/6≈5.83 |
| Qing Lu | Cox regression | Univariate + multivariate LR | 19 | 522/—/— | — | Age, ALB, ALT. | 3 | 75/3=25 |
| Wenpei Liu | LR | Univariate + multivariate Cox regression | 26 | 1010/—/— | — | RTL (measured value) + smoking history + alcohol consumption history + history of prophylactic hepatoprotective agent use + prior liver disease history + rs4387287 locus (AC+CC/AA). | 6 | 202/6≈33.67 |
| Shaoqiang Wu | LR | Univariate + multivariate LR | 13 | 133/—/— | — | ALT, AST, TBIL, ALB, GGT, LSM. | 6 | 65/6≈10.83 |
| Ying Zeng | Generalized Linear Model, Distributed Random Forest, Gradient Boosting Machine, Fully Connected Deep Neural Network, Extremely Randomized Trees, Stacked Ensemble, Extreme Gradient Boosting | Univariate + multivariate LR | 24 | 147/37/— | — | Rifampicin Cmax, BMI, total bilirubin, ALT, AST, total bile acids, creatinine. | 7 | 19/7≈2.71 |
| Quanxian Liu | Random Forest | Univariate analysis + machine learning feature importance analysis | — | 72/—/— | — | Fecal microbiome, serum/urine metabolome, serum proteome. | — | — |
| Taisei Mushiroda | LR | Importance analysis | 24 | 366/—/— | — | Age, NAT2 slow acetylator status. | 2 | 73/2=36.5 |
| Gangfeng Zhou | LR | Machine learning feature importance analysis | 14 | 1512/648/564 | — | Age ≥60 years, BMI <18.5 kg/m², alcohol use, extrapulmonary tuberculosis, albumin <35 g/L, hemoglobin <110 g/L. | 6 | 137/6≈22.83 |
| Jingru Cheng | Logistic Regression, Decision Tree, Support Vector Machine, Random Forest, Gradient Boosting Decision Tree, Extreme Gradient Boosting, and Light Gradient Boosting Machine | Importance analysis | 27 | 1649/707/923 | Multiple imputation, mode | Baseline high‑density lipoprotein cholesterol, gamma‑glutamyl transferase, triglycerides, total cholesterol, uric acid, total bilirubin, globulin, history of liver disease. | 8 | 546/8=68.25 |
| Jingwei Zhang | LR | LR | 36 | 490/256/— | Median, mode | Fever, albumin, uric acid, monocyte percentage, aspartate aminotransferase, alanine aminotransferase, rs4435111, rs3814055. | 8 | 118/8=14.75 |
| Songjun Ji | LR | Univariate + multivariate LR | 25 | 1121/492/264 | — | Age, elevated total bilirubin, neutrophil count, uric acid, alcohol intake. | 5 | — |
| Pengyu Li | Distributed random forest, generalized linear models, gradient boosting machines, extremely randomized trees, deep neural networks, stacked ensembles and XGBoost. | Univariate analysis and LASSO regression | 40 | 253/—/— | Multiple imputation, mean | CSF chloride, Total bilirubin, Blood platelet, Hypertension | 4 | 54/4=13.5 |

**Note:** LR = Logistic regression; LASSO = LASSO regression; D = model development; I = internal validation; E = external validation; — indicates not reported; N indicates unprocessed variables. BMI = Body Mass Index; ALT = Alanine Aminotransferase; INR = International Normalized Ratio; UA = Uric Acid; HGB = Hemoglobin; PLT = Platelet count; AST = Aspartate Aminotransferase; DBIL = Direct Bilirubin; CYP2E1 = Cytochrome P450 family 2 subfamily E member 1; NAT2 = N‑acetyltransferase 2; MDH = Malate Dehydrogenase; GGT = Gamma‑glutamyl transferase; EOS = Eosinophil count; PCT = Procalcitonin; PLT = Platelet (count); MLR = Monocyte‑to‑Lymphocyte Ratio; FIB = Fibrinogen; HGB = Hemoglobin; BUN = Blood Urea Nitrogen; TBA = Total Bile Acids; ALP = Alkaline Phosphatase; ChE = Serum Cholinesterase; PA = Prealbumin; CRP = C‑reactive Protein; CD4/CD8 = CD4+ T cells / CD8+ T cells; RTL = Relative Telomere Length; LSM = Liver Stiffness Measurement; HDLC = Baseline high‑density lipoprotein cholesterol; TCHOL = Total Cholesterol; TBIL = Total Bilirubin; GLB = Globulin; ALB = Albumin; NAT2 = N‑acetyltransferase 2. CSF = Cerebrospinal fluid.

**Table 3 Performance and Presentation Forms of Risk Prediction Models**

| **Study** | **Model performance** | | | **Model presentation method** | **Applicability and Limitations** |
| --- | --- | --- | --- | --- | --- |
|  | **AUC (D/I/E)** | **Calibration method** | **Specificity/Sensitivity/Ccuracy** |  |  |
| Peng Zhao | 0.749/—/— | Hosmer‑Lemeshow test, DCA | 0.752/0.640/— | Nomogram | Single‑center study, without external validation. |
| Lu Xu | Model 1: 0.851/—/—  Model 2: 0.827/—/— | — | Model 1: 0.9564/0.6176/0.9236；  Model 1: 0.9186/0.4804/0.8762 | Regression model equation | Retrospective study, without external validation. |
| Muxing Chen | 0.924/0.953/— | Calibration curve, Hosmer‑Lemeshow test, DCA | 0.80/—/0.84 | Nomogram | Single‑center study, without external validation. |
| Simin Liang | 0.833/—/— | Calibration curve | 0.876/0.732/0.608 | Nomogram | Single‑center, retrospective study with a relatively small sample size. |
| Junling Geng | Model 1: 0.803/0.791/—  Model 1: 0.726/—/— | Model 1: Calibration curve, Hosmer‑Lemeshow test, DCA; Model 2: — | Model 1: 0.733/0.742/—;  Model 1: 0.679/0.714/— | Nomogram | Single‑center, retrospective study with a relatively small sample size. |
| Xiaona Li | 0.66/—/— | — | 0.45/0.83/— | — | Primarily studying serum energy metabolism markers; single‑center, retrospective study with a small sample size. |
| Yu Guo | — | Calibration curve, DCA | — | Nomogram，Regression model equation | Single‑center, retrospective study with a relatively small sample size. |
| Leina Yao | 0.902 | Calibration curve | — | Nomogram | Single‑center, retrospective study without external validation. |
| Zhengtai Gu | 0.88/0.86/— | Calibration curve, DCA | 0.7516/0.8276/— | Web‑based nomogram | Single‑center, retrospective study without external validation. |
| Junqi He | Model 1: 0.85/—/—  Model 1: 0.866/—/—  Model 1: 0.884/—/— | Goodness‑of‑fit test | Model 1: 0.9333/0.5712/0.885  Model 1: 0.9111/0.5712/0.868  Model 1: 0.9302/0.556/0.846 | — | Retrospective study with a relatively small sample size, no external validation, and model presentation not reported. |
| Weiwei Liu | 0.822/0.737/— | Calibration curve, Hosmer‑Lemeshow test, DCA | 0.898/0.664/— | Nomogram | Single‑center, retrospective study without external validation. |
| Jinqi He | 0.681/—/— | — | —/—/0.631 | — | Retrospective study with a small sample size, no external validation, and model presentation not reported. |
| Qinqin Xie | 0.881/—/— | — | —/—/0.951 | Nomogram | Single‑center, retrospective study without external validation. |
| Jingyuan Feng | Model 1: 1/0.9935/—  Model 1: 1/0.9938/— | — | 1/1/1 | — | Different predictive models were established based on the presence or absence of concurrent hepatitis B; relatively large sample size; retrospective study; no external validation; model presentation not reported. |
| Yue Cheng | 0.842/—/— | Calibration curve, Hosmer‑Lemeshow test | — | Nomogram | Single‑center, retrospective study with a small sample size and no external validation. |
| Qing Lu | 0.725/—/— | — | — | — | Single‑center, retrospective study with a small sample size, no internal or external validation, and model presentation not reported. |
| Wenpei Liu | 0.624/—/— | — | — | Regression model equation | Single‑center, retrospective study with a small sample size and no internal or external validation. |
| Shaoqiang Wu | — | — | — | Nomogram | Single‑center, retrospective study with a small sample size and no internal or external validation. |
| Ying Zeng | 0.838/0.784/— | — | 0.856/0.733/0.844 | — | Single‑center, retrospective study with a small sample size, no external validation, and model presentation not reported. |
| Quanxian Liu | 0.88/—/— | — | — | — | Multicenter study; the first to incorporate fecal microbiome research; retrospective study with a small sample size, no external validation, and the specific candidate factors and model presentation were not reported. |
| Taisei Mushiroda | — | — | — | Regression model equation | Multicenter study incorporating NAT2 genotyping; retrospective study with a small sample size and no internal or external validation. |
| Gangfeng Zhou | 0.80/0.75/0.77 | Calibration curve, Hosmer‑Lemeshow test, DCA | — | Nomogram | Multicenter; retrospective study with a relatively small sample size. |
| Jingru Cheng | 0.764/—/0.721 | — | 0.610/0.785/0.637 | — | Multicenter; retrospective study; model presentation not reported. |
| Jingwei Zhang | 0.8164/0.7189/— | Calibration curve, Hosmer‑Lemeshow test, DCA | 0.9799/0.3425/0.7813 | Nomogram，Regression model equation | Study incorporating single nucleotide polymorphisms; single‑center, retrospective study. |
| Songjun Ji | 0.833/0.668/0.753 | Calibration curve, Hosmer‑Lemeshow test, DCA | 0.805/0.759/— | Nomogram | Multicenter; retrospective study with a relatively small sample size. |
| Pengyu Li | 0.921/0.828/— | Calibration curve, DCA | — | — | Single‑center, retrospective study with a small sample size, no external validation, and model presentation not reported. |

**Note:** AUC = Area Under Curve; DCA = Decision Curve Analysis; D = model development, I = internal validation, E = external validation; — indicates not reported; NAT2 = N‑acetyltransferase 2.

**Table 4 Definition and assessment approaches for ATB-DILI**

| **Study** | **Reference Guide** | **Biochemical threshold** | **Formal Causality Assessment** | **Other etiologies excluded** | **Evaluation time point** |
| --- | --- | --- | --- | --- | --- |
| Peng Zhao | Chinese Medical Association Tuberculosis Branch.Guidelines for diagnosis and management of drug-induced liver injury caused by anti-tuberculosis drugs (2019 version) | According to guidelines: ALT > 3×ULN or TBIL > 2×ULN; and concurrently elevated AST/ALP/TBIL with at least one of these ≥ 2×ULN. | RUCAM score ≥ 3 | Exclusion of fatty liver, viral hepatitis, alcoholic liver disease, autoimmune hepatitis, etc. | During hospitalization |
| Lu Xu | Chinese Medical Association Tuberculosis Branch.Guidelines for diagnosis and management of drug-induced liver injury caused by anti-tuberculosis drugs (2019 version) + The Study of Drug Induced Liver Disease of Chinese Medical Association. Diagnosis and treatment guideline on drug-induced liver injury | According to guidelines: ALT > 3×ULN or TBIL > 2×ULN; and concurrently elevated AST/ALP/TBIL with at least one of these ≥ 2×ULN. | RUCAM score ≥ 3 | Exclusion of viral hepatitis, alcoholic liver disease, fatty liver disease, autoimmune hepatitis, etc. | During hospitalization |
| Muxing Chen | Chinese guideline for the diagnosis and treatment of drug-induced liver injury：an update | ALT ≥ 5×ULN; ALP ≥ 2×ULN; or ALT ≥ 3×ULN with TBIL ≥ 2×ULN. | RUCAM score ≥ 3 | Exclusion of patients with pre-existing liver disease and abnormal liver function tests. | Within 3 months before treatment |
| Simin Liang | Chinese Medical Association Tuberculosis Branch.Guidelines for diagnosis and management of drug-induced liver injury caused by anti-tuberculosis drugs (2019 version) | ALT > 3×ULN or TBIL > 2×ULN; and concurrently elevated AST/ALP/TBIL with at least one of these ≥ 2×ULN. | RUCAM score ≥ 3 | Exclusion of other causes of abnormal liver function. | During treatment |
| Junling Geng | Chinese Medical Association Tuberculosis Branch.Guidelines for diagnosis and management of drug-induced liver injury caused by anti-tuberculosis drugs (2019 version) | ALT > 3×ULN or TBIL > 2×ULN; and concurrently elevated AST/ALP/TBIL with at least one of these ≥ 2×ULN. | RUCAM score ≥ 3 | Exclusion of pre-treatment abnormal liver function, malignancy, etc. | During treatment |
| Xiaona Li | Chinese Medical Association Tuberculosis Branch.Guidelines for diagnosis and management of drug-induced liver injury caused by anti-tuberculosis drugs (2019 version) | ALT > 3×ULN or TBIL > 2×ULN; and concurrently elevated AST/ALP/TBIL with at least one of these ≥ 2×ULN. | No | Not clearly listed. | Follow-up for 8 months |
| Yu Guo | No | Symptomatic ALT/AST ≥ 3×ULN; symptomatic TBIL ≥ 2×ULN; or asymptomatic ALT/AST/TBIL ≥ 5×ULN. | No | Exclusion of previous history of liver injury and diseases affecting liver or kidney function, etc. | Observed for 60 days |
| Leina Yao | No | Unclear | RUCAM score ≥ 3 | Exclusion of severe impairment of other organs. | Not specified |
| Zhengtai Gu | Chinese Medical Association Tuberculosis Branch. Guidelines for the diagnosis and treatment of drug-induced liver injury caused by antituberculosis drugs (2024 edition) | ALT ≥ 3×ULN or TBIL ≥ 2×ULN; and concurrently elevated AST/ALP/TBIL with at least one of these ≥ 2×ULN. | No | Exclusion of other causes of abnormal liver function. | Follow-up until treatment completion |
| Junqi He | No | Two consecutive measurements of ALT or TB-DILL exceeding the upper limit of normal. | No | Exclusion of hepatitis B, liver cirrhosis, etc. | After treatment |
| Weiwei Liu | Chinese Medical Association Tuberculosis Branch.Guidelines for diagnosis and management of drug-induced liver injury caused by anti-tuberculosis drugs (2019 version) | According to guidelines: ALT > 3×ULN or TBIL > 2×ULN; and concurrently elevated AST/ALP/TBIL with at least one of these ≥ 2×ULN. | No | Exclusion of abnormal liver function caused by viral hepatitis, alcoholic liver disease, fatty liver disease, autoimmune hepatitis, etc. | During treatment |
| Jinqi He | Chinese Medical Association Tuberculosis Branch.Guidelines for diagnosis and management of drug-induced liver injury caused by anti-tuberculosis drugs (2019 version) | According to guidelines: ALT > 3×ULN or TBIL > 2×ULN; and concurrently elevated AST/ALP/TBIL with at least one of these ≥ 2×ULN. | No | Exclusion of liver injury that had occurred before medication administration. | 3–4 weeks after medication initiation |
| Qinqin Xie | Chinese Medical Association Tuberculosis Branch.Guidelines for diagnosis and management of drug-induced liver injury caused by anti-tuberculosis drugs (2019 version) | ALT > 3×ULN or TBIL > 2×ULN; and concurrently elevated AST/ALP/TBIL with at least one of these ≥ 2×ULN. | RUCAM scale (score not specified) | Exclusion of pre-treatment abnormal liver function. | During hospitalization |
| Jingyuan Feng | Chinese Medical Association Tuberculosis Branch. Guidelines for the diagnosis and treatment of drug-induced liver injury caused by antituberculosis drugs (2024 edition) | Symptomatic ALT ≥ 3×ULN; asymptomatic ALT ≥ 5×ULN; TBIL ≥ 2×ULN with elevated ALT/ALP; mixed type: ALT ≥ 3×ULN with ALP ≥ 2×ULN; or INR ≥ 1.5 with hyperbilirubinemia. | RUCAM scale (score not specified) | Exclusion of liver injury due to other causes, such as autoimmune hepatitis, hepatic malignancy, and baseline abnormal liver function. | Enrolled 4 weeks after treatment initiation |
| Yue Cheng | Chinese Medical Association Tuberculosis Branch. Guidelines for the diagnosis and treatment of pulmonary tuberculosis. (2001 version) | ALT ≥ 2×ULN. | No | Exclusion of other drugs causing abnormal liver function, as well as concurrent liver cirrhosis, lung cancer, etc. | Recorded by weeks after treatment |
| Qing Lu | Chinese Medical Association Tuberculosis Branch.Guidelines for diagnosis and management of drug-induced liver injury caused by anti-tuberculosis drugs (2019 version) | ALT > 3×ULN or TBIL > 2×ULN; and concurrently elevated AST/ALP/TBIL with at least one of these ≥ 2×ULN. | RUCAM score not assessed in some cases | Exclusion of underlying liver diseases such as hepatitis, liver cirrhosis, and hepatocellular carcinoma. | Follow-up until 24 weeks |
| Wenpei Liu | Chinese Medical Association Tuberculosis Branch.Guidelines for diagnosis and management of drug-induced liver injury caused by anti-tuberculosis drugs (2019 version) | ALT > 3×ULN or TBIL > 2×ULN; and concurrently elevated AST/ALP/TBIL with at least one of these ≥ 2×ULN. | RUCAM score ≥ 3 | Exclusion of abnormal liver function test results caused by other factors. | During the treatment course |
| Shaoqiang Wu | International DILI Expert Working Group | Symptomatic ALT/AST > 3×ULN; or asymptomatic ALT/AST > 5×ULN with TBIL > 2×ULN. | No | Exclusion of acute liver failure, hepatocellular carcinoma, HIV co-infection, and previous drug-induced liver injury from other medications. | Throughout the entire treatment course |
| Ying Zeng | CSH guidelines for the diagnosis and treatment of drug-induced liver injury + Guidance for the clinical evaluation of traditional Chinese medicine-induced liver injuryIssued by China food and  drug administration + Guideline for primary care of drug-induced liver injury: practice version (2019) | Grade 1: reversible elevation of ALT and/or ALP, TB < 2.5×ULN, INR < 1.5; Grade 2: elevated ALT and/or ALP, TB ≥ 2.5×ULN or INR ≥ 1.5; Grade 3: elevated ALT and/or ALP, TB ≥ 5×ULN or INR ≥ 1.5; Grade 4: elevated ALT and/or ALP, TB ≥ 10×ULN or daily increase ≥ 1.0 mg/dL, INR ≥ 2.0 or PTA < 40%; Grade 5: fatal ATB-DILI or requiring liver transplantation. | No | Exclusion of pre-enrollment liver dysfunction, concurrent viral hepatitis, fatty liver disease, Epstein-Barr virus infection, and cytomegalovirus infection. | Intensive phase (2–3 months) |
| Quanxian Liu | National Health and Family Planning Commission of the People's Republic of China. Diagnostic criteria for tuberculosis (WS 288-2017) | ALT ≥ 2×ULN or TBIL ≥ 2×ULN. | RUCAM score ≥ 3 | Exclusion of baseline abnormal liver function, liver disease, and hepatotoxic medications. | Every 2 weeks during the first 2 months of treatment |
| Taisei Mushiroda | World Health Organization toxicity classification standards | AST/ALT/TBIL > 3×ULN. | No | Not clearly listed. | During treatment |
| Gangfeng Zhou | EASL Clinical Practice Guidelines. Drug-induced liver injury + An official ATS statement: Hepatotoxicity of antituberculosis therapy | ALT ≥ 3×ULN (with compatible symptoms); ALT ≥ 5×ULN (without symptoms); ALP ≥ 2×ULN (in the absence of bone pathology). | No | Exclusion of baseline liver disease, baseline use of hepatoprotective agents, baseline concurrent use of other non-anti-tuberculosis hepatotoxic drugs, HIV co-infection, and malignancy. | During treatment |
| Jingru Cheng | Case definition and phenotype standardization in drug-induced liver injury + Drug-induced liver injury: Asia Pacific Association of Study of Liver consen sus guidelines. Hepatol Int. | ALT ≥ 3×ULN and/or TBIL ≥ 2×ULN; and concurrently elevated AST/ALP/TBIL with at least one of these ≥ 2×ULN. | RUCAM score ≥ 3 | Exclusion of tumor, HIV/AIDS, syphilis, underlying liver disease, etc. | During treatment |
| Jingwei Zhang | WHO. Common Terminology Criteria for Adverse Events (CTCAE) Version 5.0. U.S. Department of Health and Human Services | According to guidelines: any one of ALT > ULN, AST > ULN, ALP > ULN, or TBIL > ULN. | No | Exclusion of tumor, HIV/AIDS, syphilis, underlying liver disease, psychiatric disorders, and pre-treatment abnormal liver function parameters. | During treatment |
| Songjun Ji | No | ALT ≥ 3×ULN; TBIL ≥ 2×ULN; and concurrently elevated AST/ALP/TBIL with at least one of these ≥ 2×ULN. | No | Exclusion of baseline liver disease (hepatitis B/hepatitis C/fatty liver disease), baseline therapeutic or prophylactic use of hepatotoxic agents, concurrent HIV infection, and hepatocellular carcinoma. | Within 180 days after treatment |
| Pengyu Li | Chinese Medical Association Tuberculosis Branch (2024). Guidelines for diagnosis and management of drug-induced liver injury caused by anti-tuberculosis drugs (2024 version). | ALT ≥ 3×ULN and/or TBIL ≥ 2×ULN; and concurrently elevated AST/ALP/TBIL with at least one of these ≥ 2×ULN. | No | Exclusion of baseline abnormal liver biochemistry, concurrent other types of meningitis, Epstein-Barr virus or cytomegalovirus infection, multiple organ dysfunction, sepsis, other severe comorbidities, and those receiving renal replacement therapy. | Within 2 months after treatment |

**Note:** ULN= Upper Limit of Normal; RUCAM = the Roussel Uclaf Causality Assessment Method; ALT = Alanine Aminotransferase; AST = Aspartate Aminotransferase; DBIL = Direct Bilirubin; ALP = Alkaline Phosphatase.

**Table 5 Summary Table of Predictive Factor Definitions**

| Predictor | Author, year | OR | Low | High | Definition |
| --- | --- | --- | --- | --- | --- |
| History of liver disease | Peng Zhao2022 | 4.961 | 2.982 | 8.254 | categorical variables |
|  | Lu Xu2023 | 4.072 | 3.182 | 5.212 | categorical variables |
|  | Muxing Chen2024 | 6.64 | 1.72 | 25.66 | categorical variables |
|  | Siming Liang2023 | 3.501 | 1.032 | 10.969 | categorical variables |
|  | Leina Yao2022 | 1.619 | 1.126 | 2.329 | categorical variables |
|  | Zhengtai Gu2025 | 2.07 | 1.05 | 4.1 | categorical variables |
|  | Weiwei Liu2025 | 8.737 | 4.075 | 18.455 | categorical variables |
|  | Yue Cheng2026 | 3.884 | 1.436 | 10.507 | categorical variables |
|  | Wenpei Liu2022 | 1.411 | 0.925 | 2.152 | categorical variables |
| Extrapulmonary tuberculosis | Peng Zhao2022 | 1.876 | 1.403 | 2.508 | categorical variables |
|  | Muxing Chen2024 | 3.73 | 1.87 | 7.45 | categorical variables |
|  | Siming Liang2023 | 3.407 | 1.187 | 9.388 | categorical variables |
|  | Junling Geng2024 | 2.153 | 1.237 | 3.749 | categorical variables |
|  | Gangfeng Zhou2025 | 2.53 | 1.81 | 4.11 | categorical variables |
| Age | Lu Xu2023 | 3.335 | 2.576 | 4.318 | categorical variables (≥60 years） |
|  | Yue Cheng2026 | 4.437 | 1.826 | 10.778 | categorical variables (≥60 years） |
|  | Qing Lu2026 | 1.028 | 1.013 | 1.044 | categorical variables (≥60 years） |
|  | Gangfeng Zhou2025 | 2.75 | 1.67 | 4.18 | categorical variables (≥60 years） |
| Alcohol consumption | Muxing Chen2024 | 3.01 | 1.27 | 7.15 | categorical variables |
|  | Weiwei Liu2025 | 8.713 | 3.138 | 26.265 | categorical variables |
|  | Yue Cheng2026 | 2.375 | 1.015 | 5.556 | categorical variables |
|  | Gangfeng Zhou2025 | 2.22 | 1.35 | 3.66 | categorical variables |
|  | Songjun Ji2023 | 3.209 | 1.52 | 6.652 | categorical variables |
| No use of hepatoprotective agents | Lu Xu2023 | 1.565 | 1.122 | 2.184 | categorical variables |
|  | Leina Yao2022 | 1.726 | 1.035 | 2.876 | categorical variables |
| Polypharmacy | Muxing Chen2024 | 2.99 | 1.25 | 7.13 | categorical variables |
|  | Leina Yao2022 | 6.203 | 2.236 | 10.033 | categorical variables |
| Retreatment | Siming Liang2023 | 2.599 | 1.175 | 6.083 | categorical variables |
|  | Yue Cheng2026 | 3.447 | 1.374 | 8.651 | categorical variables |
| Uric acid | Junling Geng2024 | 0.998 | 0.996 | 0.999 | continuous variables |
|  | Jingwei Zhang2022 | 0.997 | 0.995 | 1 | continuous variables |
|  | Songjun Ji2023 | 0.997 | 0.993 | 1 | continuous variables |
| AST | Zhengtai Gu2025 | 1.03 | 1.02 | 1.05 | continuous variables |
|  | Jingwei Zhang2022 | 1.051 | 1.004 | 1.101 | continuous variables |
| Diabetes mellitus | Leina Yao2022 | 1.933 | 1.328 | 2.812 | categorical variables |
|  | Zhengtai Gu2025 | 2.66 | 1.4 | 5.07 | categorical variables |
| Smoking | Zhengtai Gu2025 | 1.98 | 1.09 | 3.59 | categorical variables |
|  | Wenpei Liu2022 | 1.788 | 0.992 | 3.222 | categorical variables |
